# Significance of the viral load of high-risk HPV in the diagnosis and prediction of cervical lesions: a retrospective study

**DOI:** 10.1186/s12905-021-01493-0

**Published:** 2021-10-08

**Authors:** Yang Liu, Changjun Xu, Jing Pan, Chunyi Sun, Honglin Zhou, Yushi Meng

**Affiliations:** 1 Department of Reproduction, The Second Affiliated Hospital of Kunming Medical University, Kunming, China; 2 Department of Reproductive Medicine, People’s Hospital of Yuxi City, Yuxi, China; 3 Department of Gynecology, The Second Affiliated Hospital of Kunming Medical University, No.374 Yunnan-Burma Avenue, Kunming, 650101 China

**Keywords:** Cervical cancer, Cervical intraepithelial neoplasia, Human papillomavirus, Viral load, Predictive value

## Abstract

**Background:**

The significance of HPV viral load in the detection of cervical lesions is still controversial. This study analyzed the correlation between the high-risk HPV viral load and different cervical lesion degrees.

**Methods:**

This retrospective study included women positive for high-risk HPV DNA and screened for cervical lesions between 01/2015 and 06/2018. The high-risk HPV DNA load was measured by the second-generation Hybrid Capture technology and classified as low, moderate, and high. Colposcopy and biopsy were performed in all patients. The patients were grouped as normal, cervical intraepithelial neoplasia (CIN) grade 1, CIN grade 2, CIN grade 3, and cervical cancer. Multivariable logistic regression was performed to explore the association between high-risk HPV DNA load and cervical lesions. The odds ratios (ORs) represent the odds for increasing from low to high viral load.

**Results:**

Finally, 265 patients were grouped as normal (n = 125), CIN 1 (n = 51), CIN 2 (n = 23), CIN 3 (n = 46), and cervical cancer (n = 20). Among them, 139 (52.5%) had a low viral load, 90 (34.0) had a moderate viral load, and 36 (13.4%) had a high viral load. Taking the normal control group as a reference, a high viral load was an independent factor for CIN 1 (OR = 3.568, 95% CI: 1.164–10.941, *P* = 0.026), CIN 2 (OR = 6.939, 95% CI: 1.793–26.852, *P* = 0.005), CIN 3 (OR = 7.052, 95% CI: 2.304–21.586, *P* = 0.001), and cervical cancer (OR = 8.266, 95% CI: 2.120–32.233, *P* = 0.002).

**Conclusions:**

Among women who underwent cervical biopsy, higher high-risk HPV viral load in cervical lesions was associated with a higher risk of high-grade cervical lesions.

## Background

Cervical cancer is the fourth major malignant tumor in women worldwide [1]. In 2018, there were an estimated 570,000 new cases of cervical cancer in the world and 311,000 deaths, with 85% of the new cases being in developing countries [2]. In China, the incidence of cervical cancer has been increasing in recent years, but the mortality has been decreasing [3–5], probably because of the implementation of screening and early treatment of cervical lesions [6].

Human papillomavirus (HPV) infection can lead to cervical precancerous lesions and cervical cancer [7]. Active papillomavirus infection occurs when infected basal cells replicate and fill an area [8]. Persistent HPV infection results in squamous intraepithelial lesions graded as cervical intraepithelial neoplasia (CIN) 1, CIN 2, and CIN 3 according to how much epithelium is impacted [8]. Different types of HPV have different potentials of causing cervical cancer. High-risk HPV is the main cause of cervical cancer. High-risk HPV subtypes such as HPV16, 18, 31, 33, 35, 39, 45, 51, 52, 56, and 59 are closely associated (in decreasing order of oncogenic potential) with the occurrence of cervical cancer. This association is seen throughout the world [9], but there are some regional differences in the prevalence of the different HPV subtypes [10, 11]. The high-risk HPV viral load indicates the activity of HPV-DNA in the body [12, 13]. Studies showed that the type of HPV infection is closely related to the severity of cervical lesions and treatment prognosis [9–11], but there are inconsistent results among the studies on the correlation between the HPV viral load and the severity of cervical lesions [12–18]. Indeed, Xi et al. [12] reported that HPV16 viral load was associated with CIN 3 only, while Berggrund et al. [13] showed high-risk HPV viral load was associated with CIN 2 + lesions. Dong et al. [14] reported that the HPV 16/31/33/52/58 viral load was associated with cervical lesion severity. Del Rio-Ospina et al. [15] reported that a low HPV16 viral load was more associated with cervical lesions than a high HPV16 load. Shen et al. [16] reported an association between high-risk HPV viral load and cervical lesion severity. Long et al. [18] reported that HPV16/33/58 viral load was associated with premalignant cervical lesion severity. Hence, at present, the correlation between the HPV viral load and cervical lesions has not been determined, and the significance of the HPV viral load in the detection and treatment of cervical lesions is still controversial [16, 18].

Therefore, the objective of this study was to investigate the changes in high-risk HPV viral load in patients with different degrees of cervical lesions to clarify the correlation between the viral load of HPV and cervical lesions and to explore the clinical value of high-risk HPV viral load for the predictive diagnosis of cervical cancer.

## Methods

### Study design and patients

This retrospective study included patients who first visited the Gynecology Clinic of the Second Affiliated Hospital of Kunming Medical University between January 2015 and June 2018. All methods were performed in accordance with the Declaration of Helsinki and the relevant guidelines and regulations. This study was approved by the ethics committee of the Second Affiliated Hospital of Kunming Medical University. As a retrospective study, informed consent was exempted by the ethics committee of the Second Affiliated Hospital of Kunming Medical University.

The included women were women with a history of sexual activity and who spontaneously sook screening. For HPV-negative patients, re-screening for HPV in 3–5 years or anytime in the presence of symptoms was recommended. The inclusion criteria were (1) patients with positive HPV-DNA who underwent cervical cancer screening according to the latest version of the American College of Obstetricians and Gynecologists (ACOG) guidelines for cervical cancer screening and prevention practice [19], and (2) patients with complete colposcopy and pathological biopsy to determine the degree of lesions. The exclusion criteria were (1) previous positive HPV test, (2) history of treatment for cervical disease, and (3) incomplete data.

### Data collection and grouping

The age of the patients, the HPV-DNA viral load, and the pathological degree of cervical lesions at the time of screening were obtained from the clinical records. The patients were divided into five groups: normal group, CIN grade 1 group, CIN grade 2 group, CIN grade 3 group, and invasive cervical cancer group.

### Detection of HPV-DNA load

The second generation of the Hybridized Capture II gene hybridization signal amplification system from Digene Co. (Gaithersburg, MD, USA) was used to detect the content of HPV-DNA in the samples. That assay can detect 13 types of high-risk HPV subtypes simultaneously. All assays were strictly performed according to the manufacturer’s instructions. The relative light units (RLUs) and positive control values (CO) of the samples were detected. The ratio of the RLU to the CO value of the positive standard was used to indicate the viral load. RLU/CO < 1.0 was considered a negative result. RLU/PC ≥ 1.0 was considered a positive result, which means that the amount of HPV-DNA per ml of sample was > 1.0 pg. According to Lorincz et al. [20], the viral load of high-risk HPV DNA can be classified into three levels: low viral load (1.00–99.99 RLU/CO), moderate viral load (100.00–999.00 RLU/CO), and high viral load (≥ 1000.00 RLU/CO).

### Pathological results

A colposcopy biopsy was performed after the diagnosis by a specialist gynecologist. The cervix, vagina, and vulva were examined at the same time. Each quadrant of the cervix was assessed. The basic principles of direct biopsy or quadrant biopsy were followed. Multi-point biopsies were taken in the vinegar white area and iodine-stained suspected lesion area and were sent directly for pathological examination. If no abnormality were found under the microscopic examination in each quadrant, samples would be taken at the squamous columnar junction or the transformation area at 3, 6, 9 and 12 o’clock of the cervix, and a sample would be taken by cervical curettage. The specimens taken by colposcopy were submitted to the pathology department of the same hospital for histopathological examination and immunohistochemistry if necessary. The final cervical pathological diagnosis was determined after quality control by two pathologists. The results were presented according to normal (no squamous epithelial lesions), CIN grade 1, CIN grade 2, CIN grade 3, and cervical cancer.

### Statistical analysis

SPSS 24.0 (IBM, Armonk, NY, USA) was used for data processing and statistical analysis. Categorical data were presented as frequencies and percentages and analyzed using the chi-square test or Fisher’s exact test, as appropriate. The continuous data were tested for normal distribution using the Kolmogorov–Smirnov test and analyzed using the Student *t*-test or ANOVA with the LSD post hoc test. Unconditional logistic regression analysis was performed for univariable and multivariable analyses to calculate the odds ratio (OR) and 95% confidence interval (CI) for the presence of cervical lesions in different viral load levels, using the normal group as the reference. *P*-values < 0.05 were considered statistically significant.

## Results

### Characteristics of the patients

A total of 11,422 patients were screened for cervical cancer using the Hybrid Capture II system, and 1542 (13.5%) were positive for HPV. Among them, 385 patients underwent colposcopy, and then 265 underwent cervical biopsy. Therefore, 265 patients, aged 20–73, were included in this study: 125 in the normal group, 51 in the CIN grade 1 group, 23 in the CIN grade 2 group, 46 in the CIN grade 3 group, and 20 in the cervical cancer group. The patients in the cervical cancer group were significantly older than the patients in the normal, CIN grade 1, and CIN grade 2 groups (all *P* < 0.05) (Table 1).

**Table 1:** Characteristics of the patients

Characteristic, n (%)	Normal group (n = 125)	CIN I group (n = 51)	CIN II group (n = 23)	CIN III group (n = 46)	Cervical cancer (n = 20)	*P*
Age (years old), median (IQR)	38 (30, 47)*	40 (29.5, 46)*	34 (29, 40.5)*	44 (34, 50)	50 (42.5, 54.5)	< 0.001

^*^P < 0.05 versus the cervical cancer group

*CIN* Cervical intraepithelial neoplasia, *IQR* interquartile range

### HPV-DNA loads and correlation with pathological types

Among the 265 patients, 139 (52.5%) had a low viral load, 90 (34.0) had a moderate viral load, and 36 (13.4%) had a high viral load. There were significant differences in viral load among the five pathological groups (*P* < 0.001). The viral load of patients in the CIN grade 3 and cervical cancer groups was higher than that of patients in the normal group, but there were no significant differences among the other groups (Table 2).

**Table 2:** Viral load of HPV-DNA in groups with different pathological results

HPV-DNA load	Normal group (n = 125)	CIN I group (n = 51)	CIN II group (n = 23)	CIN III group (n = 46)	Cervical cancer (n = 20)	*P*
HPV-DNA viral load RLU/CO, Median (IQR)	48.52 (4.16, 180.87)	156.34 (10.54, 578.08)	159.13 (36.19, 701.465)	266.87 (16.56, 958.13)*	307.005 (72.76, 1386.075)*	< 0.001
Low load group, n (%)	82 (65.6%)	24 (47.1%)	10 (43.5%)	16 (34.8%)*	7 (35%)*	< 0.001
Medium load group, n (%)	36 (28.8%)	19 (37.2%)	8 (34.8%)	20 (43.5%)	7 (35%)	–
High load group, n (%)	7 (5.6%)	8 (15.7%)	5 (21.7%)	10 (21.7%)	6 (30%)	–

^*^P < 0.05 versus the normal group

*CIN* Cervical intraepithelial neoplasia, *IQR* interquartile range

Taking the normal control group as a reference, the correlations between all cervical lesions and viral load were analyzed and compared using multivariable analyses. The results showed that the viral load was an independent risk factor for the occurrence of CIN grade 1 and CIN grade 2 (CIN I: high load, OR = 3.568, 95% CI: 1.164–10.941, *P* = 0.026; CIN II: high load, OR = 6.939, 95% CI: 1.793–26.852, *P* = 0.005), but age was not an independent factor (all age groups *P* > 0.05). For CIN grade 3, age still was not an independent factor, but a moderate load of HPV (OR = 2.852, 95% CI: 1.322–6.152, *P* = 0.008) and a high load of HPV (OR = 7.052, 95% CI: 2.304–21.586, *P* = 0.001) were independent risk factors. For cervical cancer, age was not associated, while high-load HPV (OR = 8.266, 95% CI: 2.120–32.233, *P* = 0.002) were both independent risk factors (Table 3).

**Table 3:** Correlation between pathological types and HPV-DNA load

	CIN I				CIN II			
	Univariable OR (95% CI)	*P*	Multivariable OR (95% CI)	*P*	Univariable OR (95% CI)	*P*	Multivariable OR (95% CI)	*P*
Age < 30 years	Ref		Ref		Ref		Ref	
30–40 years	0.488 (0.187, 1.274)	0.143	0.51 (0.193, 1.345)	0.173	1.057 (0.343, 3.256)	0.923	1.173 (0.371, 3.71)	0.786
≥ 40 years	0.966 (0.432, 2.158)	0.932	0.931 (0.412, 2.107)	0.864	0.523 (0.16, 1.71)	0.283	0.483 (0.144, 1.62)	0.238
Low load	Ref		Ref		Ref		Ref	
Moderate load	1.803 (0.879, 3.698)	0.108	1.815 (0.882, 3.737)	0.106	1.822 (0.664, 4.998)	0.244	1.804 (0.654, 4.976)	0.254
High load	3.905 (1.285, 11.869)	0.016	3.568 (1.164, 10.941)	0.026	5.857 (1.561, 21.973)	0.009	6.939 (1.793, 26.852)	0.005

	CIN III				Cervical cancer			
	Univariable OR (95% CI)	*P*	Multivariable OR (95% CI)	*P*	Univariable OR (95% CI)	*P*	Multivariable OR (95% CI)	*P*
Age < 30 years	Ref		Ref		Ref		Ref	
30–40 years	1.776 (0.572, 5.514)	0.321	1.92 (0.602, 6.124)	0.271	1.902 (0.188, 19.279)	0.586	2.122 (0.206, 21.864)	0.527
≥ 40 years	2.421 (0.838, 6.99)	0.102	2.275 (0.768, 6.734)	0.138	7.172 (0.903, 56.986)	0.062	6.599 (0.818, 53.229)	0.076
Low load	Ref		Ref		Ref		Ref	
Moderate load	2.847 (1.324, 6.121)	0.007	2.852 (1.322, 6.152)	0.008	2.278 (0.744, 6.971)	0.149	2.308 (0.744, 7.159)	0.148
High load	7.321 (2.426, 22.093)	< 0.001	7.052 (2.304, 21.586)	0.001	10.041 (2.64, 38.19)	0.001	8.266 (2.12, 32.233)	0.002

*CIN* Cervical intraepithelial neoplasia, *OR* odds ratio, *CI* confidence interval

## Discussion

The correlation between HPV viral load and cervical lesions is poorly known, and the significance of HPV viral load in the detection and treatment of cervical lesions is still controversial [16, 18]. Therefore, this study aimed to analyze the correlation between the high-risk HPV viral load and different cervical lesion degrees and the value of high-risk HPV viral load in the early diagnosis and prediction of cervical lesions. The results suggest that cervical lesions are closely related to high-risk HPV infection in women who underwent cervical biopsy. Higher high-risk HPV viral load in cervical lesions was associated with a higher risk of high-grade cervical lesions. Nevertheless, the high-risk HPV DNA load could be used for triage [14].

In this population, the rate of HPV positivity among the screened women was 13.5%, which is a little lower than the national rate in China [21]. The patients with CIN grade 3 and cervical cancer were older than those with normal results, CIN grade 1, and CIN grade 2, which is consistent with the literature, i.e., that age is a risk factor for advanced cervical lesions [1, 4, 8, 19].

Since integrating the HPV DNA into the host cells is necessary for malignant transformation by the E6 and E7 proteins, the HPV DNA load has been suggested to be used as a marker of the risk of dysplasia and cervical cancer [16, 22]. In addition, the quantification of the DNA load could have a direct relationship with the risk of cervical lesions [23–25]. Clinical trials by Ronco et al. [26, 27] showed that liquid-based cytology, high-risk HPV detection using Hybrid Capture II, and biopsy in the presence of atypic squamous cells or worse was an appropriate screening strategy for cervical lesions. Many HPV infections will not lead to cervical lesions since a persistent infection is necessary for malignant transformation, and many infections are self-resolving [28]. Therefore, high viral loads should suggest persistent infections, and high viral loads indicate a lower possibility of self-resolution [29]. Hildesheim et al. [30] showed that a viral load threshold of 10 pg/mL indicated persisting HPV infection. Nevertheless, this association is still controversial [20, 31]. Lorincz et al. [20] reported no association between the viral load of 13 high-risk HPVs and the risk of CIN grade 3 and cervical cancer, while Wu et al. [23] showed that the HPV-18 viral load was low in precancerous lesions but high in cervical cancer. Ronco et al. [27] showed that HPV DNA > 2 pg/ml was associated with the presence of CIN 2+, but that triage or repeat testing was necessary since some CIN lesions can regress. The present study supports the hypothesis that high-risk HPV viral loads are associated with more advanced cervical lesions. This view is supported by Long et al. [18], who showed that the viral load of HPV-16, HPV-58, and HPV-33 was associated with high-grade cervical lesions, as well as by other studies in various populations [12–18, 32–34]. Berggrund et al. [13] showed that the high-risk HPV viral load could indicate the course of the infection and the presence of CIN grade 2, CIN grade 3, and cervical cancer. A previous study also correlated the Hybrid Capture II viral load and CIN grade [35]. Wang et al. [36] reported that single or multiple infections with HPV16/18/33/51/52/58 were associated with the risk of cervical lesions. Nevertheless, a study suggested that a single measure of HPV viral load could not reliably indicate the presence of CIN [37], and a recent study supports double-testing using the Pap test and high-risk HPV detection [38]. Luo et al. [35] suggested that a threshold of 10 RLU/CO should prompt immediate colposcopy, while 1 RLU/CO should prompt reflex testing. Therefore, future studies could consider performing serial measurements and different screening strategies.

This study has limitations. It was performed at a single center, and the sample size was relatively small, mainly because not all patients underwent Hybrid Capture II analysis. The data that could be analyzed were limited to those available in the medical charts. The exact HPV subtype was not available for many patients indicated as positive in their chart. Cytology results were not available because cytology was often performed at another lower-level hospital or clinic. No follow-up data were available. Additional studies are still necessary to refine our understanding of the relationship between HPV DNA load and cervical lesions. Since only high-risk HPV-positive patients were included, the frequency of high-risk HPV positivity in each pathological group could be analyzed.

## Conclusions

In conclusion, in the light of the previous literature, the results suggest that cervical lesions are closely related to high-risk HPV infection in women who underwent biopsy. Higher high-risk HPV viral load in cervical lesions was associated with a higher risk of high-grade cervical lesions. Therefore, high-risk HPV viral load could be used as a marker of the risk of finding high-grade cervical lesions at biopsy.

## Data Availability

The datasets used and/or analyzed during the current study are available from the corresponding author on reasonable request.

## Abbreviations

HPV: Human papillomavirus
CIN: Cervical intraepithelial neoplasia
ACOG: American College of Obstetricians and Gynecologists
RLUs: Relative light units
CO: Control values
OR: Odds ratio
CI: Confidence interval
